# Preserved ictal responsiveness in right mesial temporal lobe epilepsy: metabolic correlates in posterior temporal networks with FDG-PET

**DOI:** 10.3389/fneur.2025.1690510

**Published:** 2025-10-23

**Authors:** Yi-Seul Choo, Young-Min Shon, Seung Hwan Moon, Jeongsik Kim, Hea Ree Park, Eun Yeon Joo, Dae Won Seo

**Affiliations:** ^1^Department of Neurology, Samsung Medical Center, Sungkyunkwan University School of Medicine, Seoul, Republic of Korea; ^2^Department of Nuclear Medicine and Molecular Imaging, Samsung Medical Center, Sungkyunkwan University School of Medicine, Seoul, Republic of Korea

**Keywords:** right mesial temporal lobe epilepsy, automatisms with preserved responsiveness (APR), FDG-PET, posterior temporal networks, consciousness

## Abstract

**Objective:**

Automatisms with preserved responsiveness (APR) represent a distinctive clinical feature in right mesial temporal lobe epilepsy (MTLE). This study aimed to investigate whether interictal FDG-PET hypometabolism correlates with impaired responsiveness during seizures.

**Methods:**

We retrospectively analyzed 49 patients with right MTLE who underwent presurgical evaluation. Patients were stratified into APR+ (*n* = 16) and APR- (*n* = 33) groups based on a standardized four-domain assessment (orientation, memory, verbal command, motor execution). Interictal FDG-PET hypometabolism was visually scored on a three-point scale (0 = absent, 1 = moderate, 2 = severe) across 31 predefined brain regions by three blinded epileptologists. Group comparisons were performed using independent-sample *t* tests or Mann–Whitney U tests, with Bonferroni correction (*p* < 0.0016) and effect size estimation (Cohen’s d).

**Results:**

APR- patients exhibited significantly greater hypometabolism in the posterior lateral temporal cortex (*p* = 0.00061, Cohen’s d = 1.20) compared to APR+ patients. Although hypometabolic trends were also observed in posterior mesial (*p* = 0.00203) and posterior basal temporal regions (*p* = 0.00328), these did not survive multiple-comparison correction. No significant group differences were found in anterior temporal, frontal, insular, parietal, occipital, or subcortical regions. Contralateral hemispheric metabolism was preserved across all regions.

**Conclusion:**

Consciousness impairment in right MTLE is specifically associated with posterior lateral temporal dysfunction, with broader posterior temporal vulnerability suggested by subthreshold trends. These findings identify posterior lateral temporal hypometabolism as a potential biomarker of impaired responsiveness in right MTLE and highlight the value of FDG-PET for characterizing consciousness-related network dysfunction.

## Introduction

Automatisms with preserved responsiveness (APR) represent a unique clinical phenomenon in temporal lobe epilepsy (TLE), in which patients maintain the ability to interact with their environment despite exhibiting stereotyped motor behaviors. Because responsiveness is an observable and clinically accessible marker, APR has been regarded as a surrogate of preserved consciousness during seizures. Understanding the neural mechanisms underlying APR is essential for clarifying how seizure networks interact with consciousness-related circuits.

Previous studies have investigated ictal responsiveness using clinical observation or ictal EEG analysis. While these approaches have provided valuable insights, they are limited in their ability to capture structural or metabolic substrates of consciousness (1–3). Functional neuroimaging studies, particularly ictal SPECT, have suggested posterior cortical involvement in patients with impaired responsiveness (4). However, these findings remain inconsistent, descriptive, and not directly linked to specific cortical subregions. Moreover, validated behavioral instruments such as the Responsiveness in Epilepsy Scales (RES-I and RES-II) were developed only recently (1), and most earlier studies—including ours during the data collection period—relied on structured but non-standardized clinical assessments.

Despite these advances, no study to date has systematically examined interictal metabolic correlates of APR using FDG-PET in right mesial temporal lobe epilepsy (MTLE). FDG-PET provides a unique window into stable metabolic alterations that may reflect the integrity of consciousness-supporting networks (5).

The present study aimed to address this gap by comparing interictal FDG-PET metabolic patterns between patients with and without preserved responsiveness during automatisms. We hypothesized that distinct posterior temporal regions, particularly the posterior lateral temporal cortex, would demonstrate hypometabolism associated with impaired responsiveness. By investigating metabolic signatures of APR, we sought to provide novel insights into the network basis of consciousness in right MTLE.

## Methods

### Study population

We conducted a retrospective analysis of patients with right MTLE who underwent comprehensive presurgical evaluation at Samsung Medical Center, Seoul, South Korea, spanning from 2006 to 2020. The diagnosis of right MTLE was established through a multimodal diagnostic approach, integrating clinical history, detailed seizure semiology analysis, long-term video-EEG monitoring findings, and high-resolution neuroimaging data. As part of the standardized presurgical protocol, all patients underwent a comprehensive evaluation battery comprising video-EEG monitoring, high-resolution brain MRI, and FDG-PET imaging.

Hippocampal sclerosis (HS) was defined based on MRI findings of hippocampal atrophy with T2/FLAIR hyperintensity. In surgically treated patients, histopathological diagnoses were reviewed, and focal cortical dysplasia (FCD) was classified according to established pathological subtypes.

### APR classification and evaluation

Seizure semiology was analyzed by a panel of experienced epileptologists through systematic review of video-EEG recordings. Patient classification into APR + and APR- cohorts was determined based on a standardized assessment of responsiveness during ictal events. The evaluation protocol encompassed four distinct functional domains: verbal memory retention, temporal–spatial orientation, comprehension of verbal commands, and execution of motor tasks. Patients demonstrating preserved function in at least one domain were designated as APR+, while those exhibiting complete impairment across all domains were categorized as APR-. In addition, the specific tasks included recalling the patient’s own name, identifying colors or numbers for later retrieval, answering orientation questions regarding time and place, and following verbal and motor commands (e.g., clapping the instructed number of times, raising the right or left arm, or showing numbers with fingers). These four domains are conceptually aligned with those assessed by the *Responsiveness in Epilepsy Scales (RES-I and RES-II)*, although the formal instruments were not administered during the study period. Antiseizure drug tapering followed standardized clinical protocols during video-EEG monitoring.

Within the APR + cohort (*n* = 16), we implemented a detailed stratification system based on the spectrum of preserved responsiveness during seizures. This hierarchical classification yielded four distinct subgroups according to the number of preserved functional domains: Group 1 (*n* = 3, 18.75%) maintained function in a single domain, Group 2 (*n* = 6, 37.50%) in two domains, Group 3 (*n* = 5, 31.25%) in three domains, and Group 4 (*n* = 2, 12.50%) demonstrated preservation across all four domains (Table 1) (1, 4). Details on the number of seizures analyzed per patient, intra-individual stereotypy, seizure distribution, and antiseizure medication tapering are provided in Supplementary Table S1.

**Table 1:** Classification of APR + patients based on the degree of preserved responsiveness during seizures.

Group	Number of preserved response domains (among total of four)	Number of patients (%)
1	1	3 (18.75)
2	2	6 (37.50)
3	3	5 (31.25)
4	4	2 (12.50)

APR, Ictal automatisms with preserved responsiveness; Response domains consisted of four categories including verbal (word memory, orientation, and verbal command) and nonverbal (motor command) questions.

### FDG-PET imaging protocol

FDG-PET scans were conducted using a dedicated PET/CT scanner (Discovery LS or Discovery STe, GE Healthcare, Chicago, USA). All patients fasted for at least 6 h, and blood glucose levels were measured before injection of 18F-fluorodeoxyglucose (FDG), targeting <200 mg/dL for the examination. After intravenous administration of FDG (5 MBq/kg), patients rested for a 30-min distribution period. PET scanning was then performed using a continuous spiral technique from the base of skull to vertex. All patients maintained a clinical seizure-free state for at least 24 h before and during the PET imaging procedure.

**Figure 1:**
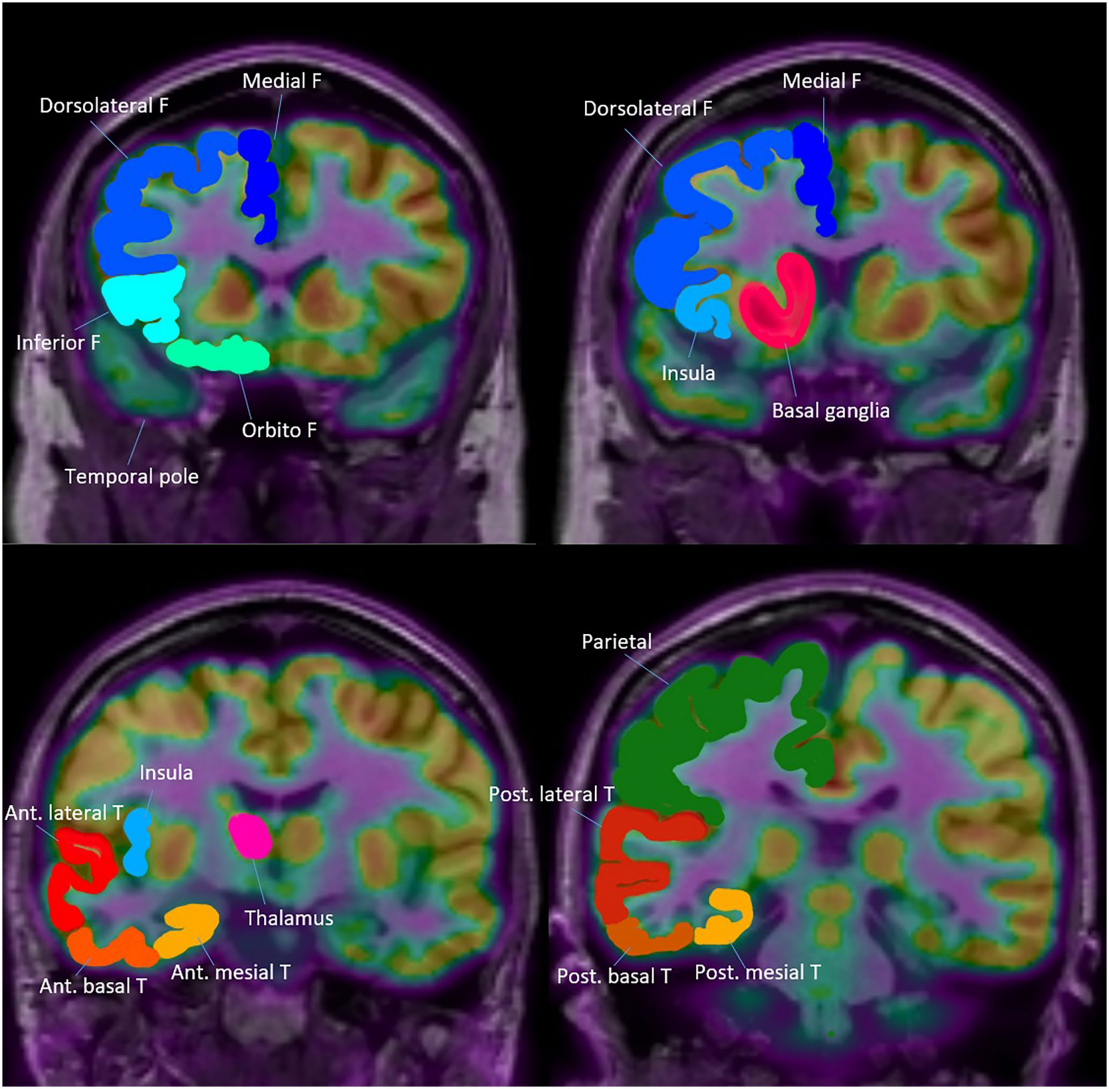
Topography of Anatomical areas assessed for regional hypometabolism on coronal FDG-PET images. This figure shows coronal schematic FDG-PET views outlining the anatomical areas evaluated for regional hypometabolism in the temporal lobe. It depicts anterior, posterior, mesial, lateral, and basal subregions that served as regions of interest in the analysis. The schematic provides spatial context for understanding how regional metabolism was assessed and compared across groups.

Visual analysis of PET images was conducted independently by three experienced epileptologists (H. B., Y. C., and Y. S.), who were blinded to all clinical data to minimize interpretation bias. The assessment of glucose metabolism followed a structured anatomical framework, encompassing both temporal and extratemporal regions (Figure 1). To ensure diagnostic accuracy and reliability, cases showing discordant interpretations among the three epileptologists and an additional imaging specialist underwent rigorous consensus review to establish definitive readings.

The temporal lobe was anatomically divided into anterior and posterior regions using the uncal apex as a landmark, with areas located anterior to the uncal apex classified as anterior and those posterior to it classified as posterior, in accordance with established anatomical criteria (5).

**Figure 2:**
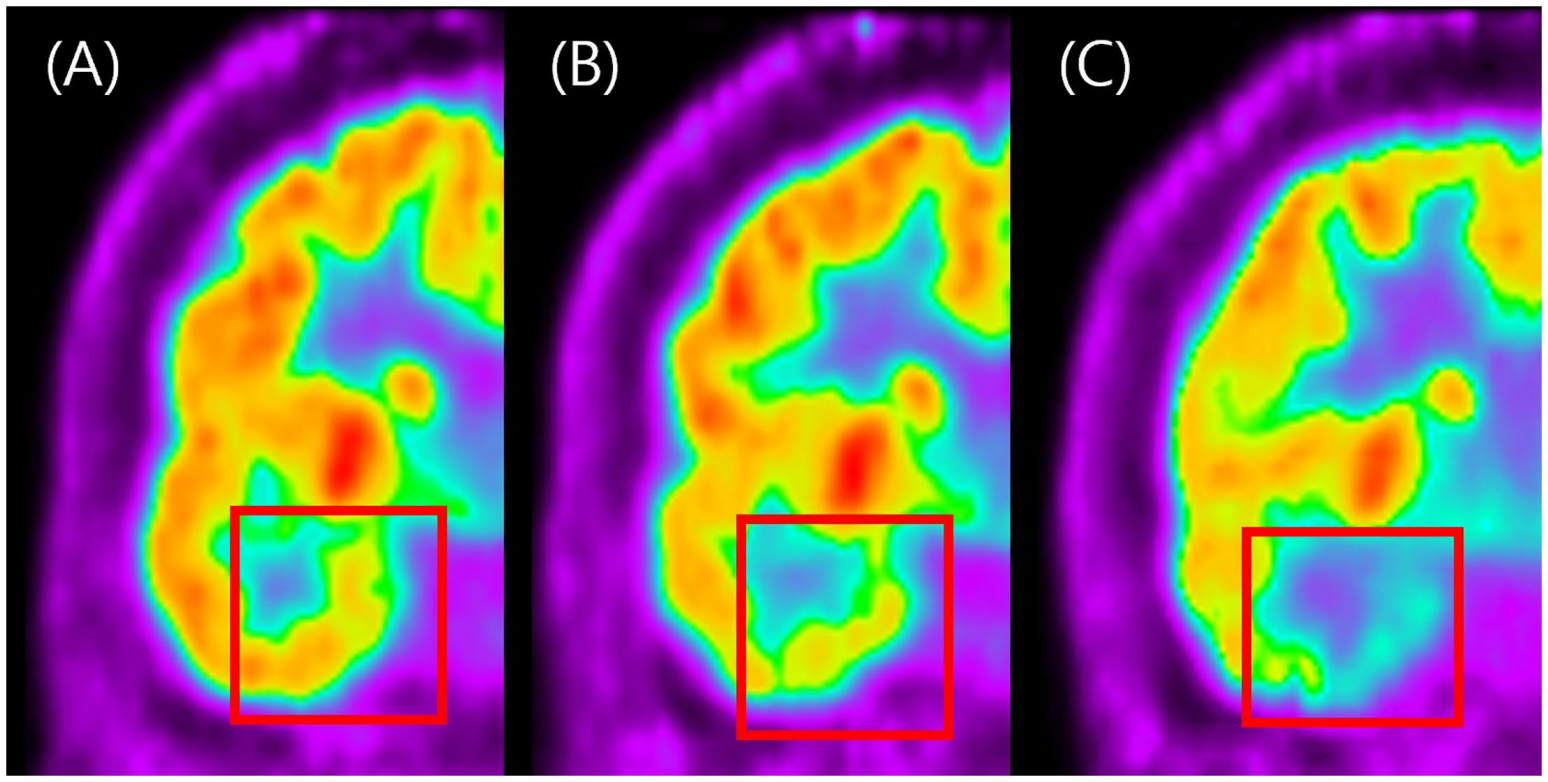
Visual grading examples of temporal hypometabolism on FDG-PET. This figure shows representative coronal FDG-PET images illustrating the three-grade visual scale used to classify the severity of temporal hypometabolism. **Panel A** represents normal symmetric metabolism with no reduction (Grade 0). **Panel B** shows mild localized reduction in metabolic activity (Grade 1), while **panel C** shows marked and extended reduction with clearly decreased FDG uptake (Grade 2). The figure demonstrates how visual grading was applied to categorize hypometabolism severity in this study.

For a more standardized visual grading process, a reference image set was developed to illustrate each severity grade, ranging from absent or minimal (Grade 0) to moderate (Grade 1) and severe (Grade 2) hypometabolism (Figure 2). The reference images were used as a standard template to ensure consistency and reliability in hypometabolism severity ratings across all anatomical regions.

### Statistical analysis

Comparative analyses of demographic and clinical characteristics between APR + and APR − cohorts were performed using appropriate statistical methods: Chi-square tests for categorical variables and Mann–Whitney U tests for non-normally distributed continuous variables. Regional FDG-PET hypometabolism was visually rated on a three-point ordinal scale (0 = minimal/absent, 1 = moderate, 2 = severe) by three independent raters blinded to clinical information. In cases of discrepancy, ratings were resolved by consensus. Group comparisons of mean visual scores were performed between APR + (*n* = 16) and APR − (*n* = 33) patients for each predefined anatomical region. Independent-sample *t* tests were used for normally distributed data, and Mann–Whitney U tests were applied otherwise. Effect sizes were calculated using Cohen’s d. To account for multiple comparisons across 31 regions, Bonferroni correction was applied (*α* = 0.0016). All statistical analyses were executed using SPSS version 18.0 (IBM Corp., Armonk, NY, USA).

## Results

### Demographics and clinical characteristics

The study cohort comprised 49 patients with right MTLE, stratified into 16 APR + and 33 APR- cases. Demographic analysis revealed comparable age distributions between groups (APR+: 33.6 ± 9.0 years; APR-: 35.9 ± 11.0 years; *p* = 0.338). Disease onset characteristics were similar, with mean age at seizure onset of 16.0 ± 9.1 years in APR + and 20.6 ± 12.6 years in APR- patients (*p* = 0.267), and disease durations of 12.2 ± 5.4 and 17.2 ± 12.7 years, respectively (*p* = 0.405). The gender distribution was balanced between groups, with males constituting 40.0% of APR + and 43.8% of APR- patients (*p* = 1.000).

Handedness and language dominance were also evaluated as part of the presurgical assessment. All patients were identified as left-hemisphere dominant for language, based on Wada test (*n* = 40) or language fMRI (*n* = 9). In addition, 46 patients (93.9%) were right-handed, supporting the classification of the right hemisphere as non-dominant in our cohort.

Neuroimaging findings demonstrated MRI-based hippocampal sclerosis in 75.8% (25/33) of APR − patients and 62.5% (10/16) of APR + patients (*p* = 0.324). Histopathological examination confirmed HS in the majority of surgical cases and identified focal cortical dysplasia type IIIa in 6/49 patients (12.2%), underscoring the heterogeneity of temporal lobe pathology in our cohort. Additional temporal lobe lesions on MRI were observed in 21.9% of APR − and 6.7% of APR + patients (*p* = 0.250).

Regarding clinical history, CNS infection was documented in 18.8% of APR + and 9.1% of APR- patients (*p* = 0.377), with no reported instances of head trauma in either group.

Treatment profiles showed comparable anti-seizure medication (ASM) burden between groups (APR+: 3.00 ± 0.82; APR-: 2.64 ± 0.90; *p* = 0.166). All patients underwent surgical intervention, with post-operative seizure freedom achieved in 62.50% (10/16) of APR + and 78.79% (25/33) of APR- patients (*p* = 0.386). Comprehensive statistical analysis revealed no significant differences in demographic or clinical parameters between the two groups (all *p* > 0.05; Table 2).

**Table 2:** Demographic and clinical characteristics of right TLE patients with APRs and without APRs.

Characteristic	APR (−) Group (*n* = 33)	APR (+) Group (*n* = 16)	*p*-value
Age	35.9 ± 11.0	33.6 ± 9.0	0.33800
Sex (Male, %)	43.8%	40.0%	1.00000
Seizure onset age	20.6 ± 12.6	16.0 ± 9.1	0.26705
Duration from seizure onset (year)	17.2 ± 12.7	12.2 ± 5.4	0.40513
Hippocampal sclerosis (%)	75.0%	60.0%	0.32400
Other temporal lesion (%)	21.9%	6.7%	0.25000
History of head trauma (%)	0.0%	0.0%	1.00000
History of CNS infection (%)	9.1%	18.8%	0.37730
Number of pre-OP ASM	2.64 ± 0.90	3.00 ± 0.82	0.16630
Number of post-OP ASM	0.21 ± 0.42	0.38 ± 0.50	0.26984
Post-OP seizure free rate (%)	78.79% (25/33)	62.50% (10/16)	0.38647

APR, Ictal automatisms with preserved responsiveness; TLE, Temporal lobe epilepsy.

Electrophysiological findings were comparable between APR- and APR + patients. Interictal epileptiform discharges (IEDs) were predominantly observed over the right frontotemporal region in both groups. The most frequently involved site was [F8 (APR-: 18/33, 54.5%; APR+: 7/16, 43.8%)], with no statistically significant difference between groups (*p* = 0.551), followed by FT8 and T8 electrodes.

Ictal EEG analysis revealed rhythmic theta-to-delta activity originating from the right temporal region in all patients, consistent with a seizure onset zone localized to the right mesial temporal lobe. These findings affirm that both APR- and APR + groups shared similar electrophysiological seizure profiles.

### FDG-PET images: regional hypometabolism analysis

FDG-PET analysis revealed distinct patterns of regional glucose metabolism between APR + and APR- patients. Quantitative comparisons of visual hypometabolism scores between APR − (*n* = 33) and APR + (*n* = 16) patients are summarized in Table 3. The most striking difference was observed in the posterior lateral temporal cortex (*p* = 0.00061, Cohen’s d = 1.20). Trends were also noted in posterior mesial temporal (*p* = 0.00203) and posterior basal temporal (*p* = 0.00328), though these did not survive Bonferroni correction (*α* = 0.0016). No contralateral regions showed significant differences after correction, suggesting ipsilateral predominance (Supplementary Table S2).

**Table 3:** Quantitative comparison of regional FDG-PET hypometabolism scores between MTLE patients with and without automatisms with preserved responsiveness.

Regional hypometabolism	APR (−) Group (*n* = 33) Mean±SD	APR (+) Group (*n* = 16) Mean±SD	Cohen’s d	*p*-value
Anterior temporal regions				
Temporal pole				
Ipsilateral	1.39 ± 0.56	1.19 ± 0.66	0.35	0.30320
Contralateral	0.36 ± 0.49	0.31 ± 0.48	0.11	0.73679
Basal temporal				
Ipsilateral	1.52 ± 0.51	1.31 ± 0.48	0.41	0.18974
Contralateral	0.73 ± 0.57	0.56 ± 0.51	0.30	0.37336
Mesial temporal				
Ipsilateral	1.79 ± 0.42	1.81 ± 0.40	−0.06	0.85463
Contralateral	1.00 ± 0.35	0.88 ± 0.50	0.31	0.31165
Lateral temporal				
Ipsilateral	1.39 ± 0.50	1.00 ± 0.52	0.78	0.02046
Contralateral	0.30 ± 0.47	0.19 ± 0.40	0.26	0.40302
Posterior temporal regions				
Basal temporal				
Ipsilateral	1.45 ± 0.51	0.94 ± 0.57	0.98	0.00523
Contralateral	0.67 ± 0.60	0.56 ± 0.51	0.18	0.62588
Mesial temporal				
Ipsilateral	1.64 ± 0.49	1.12 ± 0.50	1.04	0.00251
Contralateral	0.85 ± 0.51	0.44 ± 0.51	0.81	0.01238
Lateral temporal				
Ipsilateral	1.21 ± 0.48	0.62 ± 0.50	1.20	**0.00061**
Contralateral	0.18 ± 0.46	0.00 ± 0.00	0.47	0.10862
Extratemporal regions				
Medial frontal				
Ipsilateral	0.88 ± 0.65	0.75 ± 0.45	0.22	0.55689
Contralateral	0.67 ± 0.54	0.62 ± 0.50	0.08	0.85065
Dorsolateral frontal				
Ipsilateral	0.85 ± 0.44	0.62 ± 0.50	0.48	0.12312
Contralateral	0.09 ± 0.44	0.38 ± 0.50	−0.77	0.01790
Orbitofrontal				
Ipsilateral	1.03 ± 0.53	1.00 ± 0.52	0.06	0.85789
Contralateral	0.52 ± 0.51	0.44 ± 0.51	0.15	0.62246
Inferior frontal				
Ipsilateral	1.12 ± 0.42	1.12 ± 0.34	−0.01	1.00000
Contralateral	0.39 ± 0.56	0.44 ± 0.51	−0.08	0.69719
Insula				
Ipsilateral	1.06 ± 0.66	1.12 ± 0.34	−0.11	0.78671
Contralateral	0.45 ± 0.51	0.50 ± 0.52	−0.09	0.77673
Thalamus				
Ipsilateral	0.18 ± 0.39	0.31 ± 0.60	−0.28	0.53230
Contralateral	0.00 ± 0.00	0.00 ± 0.00	N/A	1.00000
Basal ganglia				
Ipsilateral	0.12 ± 0.33	0.25 ± 0.45	−0.35	0.26467
Contralateral	0.00 ± 0.00	0.06 ± 0.25	−0.44	0.16373
Parietal				
Ipsilateral	1.18 ± 0.53	1.00 ± 0.52	0.35	0.26467
Contralateral	0.61 ± 0.56	0.50 ± 0.52	0.20	0.56559
Occipital				
Ipsilateral	0.09 ± 0.29	0.00 ± 0.00	0.38	0.22769
Contralateral	0.03 ± 0.17	0.00 ± 0.00	0.21	0.51389

Values are mean ± standard deviation of visual hypometabolism scores (0 = minimal/absent, 1 = moderate, 2 = severe). Group comparisons were conducted between APR− and APR+ patients using independent-sample *t* tests or Mann–Whitney U tests, as appropriate. Effect sizes are reported as Cohen’s d. Bonferroni corrected significance threshold: *p* < 0.0016. Statistically significant results after correction are shown in bold. Effect sizes are expressed as Cohen’s d (d = μ_APR−−μ_APR+), where a positive value indicates greater hypometabolism in the APR- group compared to the APR+ group.

While the posterior temporal region exhibited a gradient of metabolic alterations, only the lateral component reached statistical significance after Bonferroni correction (*p* = 0.00061). Notable hypometabolic trends were observed in other posterior temporal regions (posterior mesial temporal: *p* = 0.00203; posterior basal temporal: *p* = 0.00328), though these differences did not survive Bonferroni correction. Notably, the contralateral hemisphere did not show any significant between-group differences across regions (*p* values 0.017–0.800), and no significant hypometabolism was observed in the contralateral thalamus.

**Figure 3:**
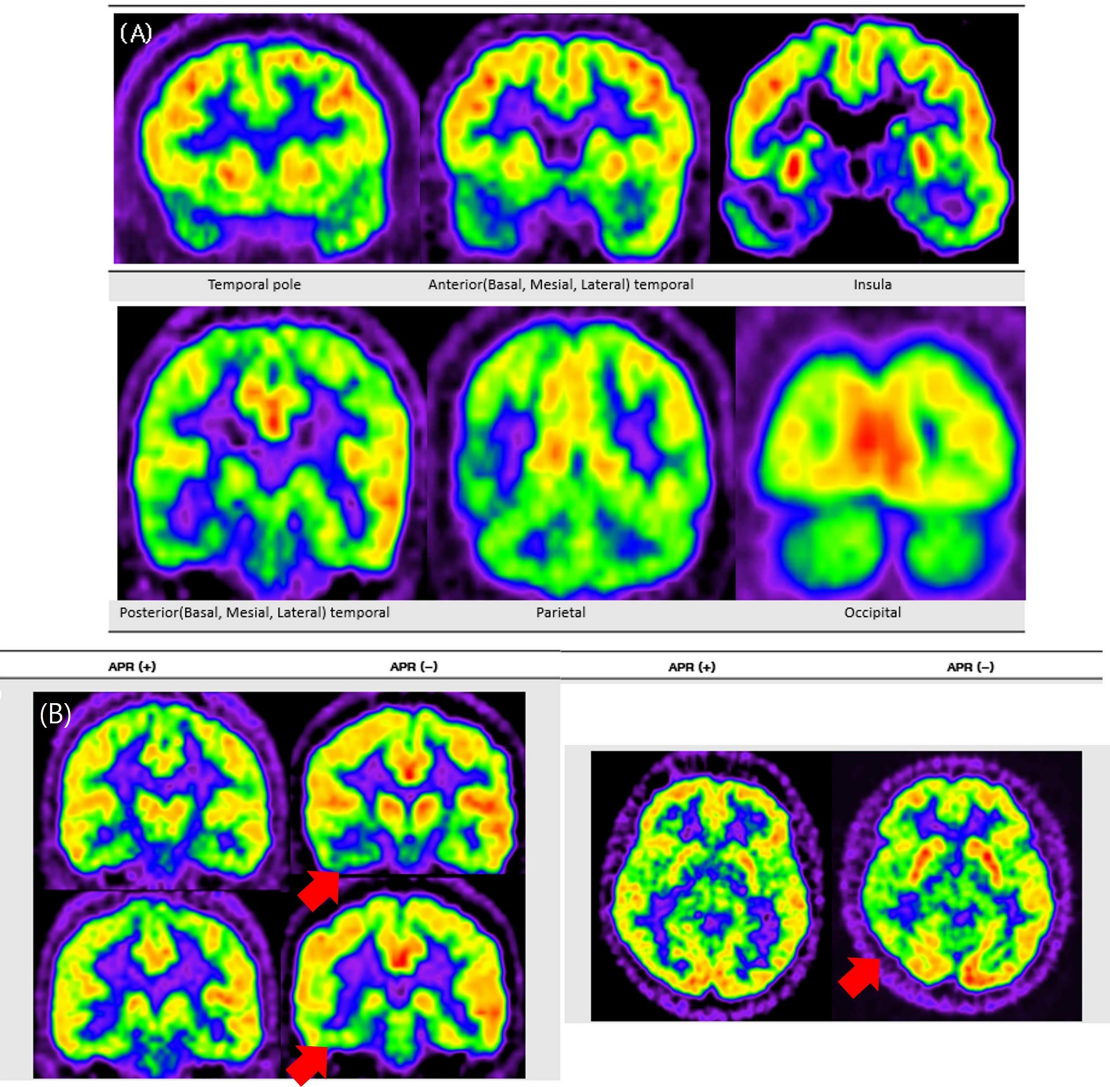
Representative FDG-PET images contrasting APR− and APR+ patients. This figure presents FDG-PET images comparing patients with and without preserved ictal responsiveness (APR+ and APR−). **Panel A** shows a typical pattern of extensive hypometabolism in a representative patient with right mesial temporal lobe epilepsy, involving the temporal pole and extending to temporal, parietal, insular, and occipital regions. **Panel B** shows two patient examples, where red arrows indicate more pronounced ipsilateral posterior temporal hypometabolism in the APR− patient compared to the APR+ patient.

Figure 2 illustrates the standardized three-point visual grading scale used in this analysis, whereas Figure 3 presents representative FDG-PET images contrasting APR + and APR − patients. These representative images clearly demonstrate more pronounced hypometabolism in posterior temporal regions of APR- patients, while other areas showed comparable metabolic activity between groups. This distinctive topographic pattern of metabolic alterations substantiates the specific association between posterior temporal hypometabolism, particularly in the lateral region, and impaired responsiveness during seizures in right MTLE patients.

## Discussion

This study demonstrates that impaired responsiveness in right MTLE is specifically associated with hypometabolism in the posterior lateral temporal cortex. This region was the only region to survive correction for multiple comparisons (*p* = 0.00061), underscoring its potential role as a key hub in the network pathophysiology of consciousness impairment (2, 8).

The posterior lateral temporal cortex is strategically positioned as an associative relay, richly interconnected with thalamo-cortical and parieto-cortical networks (9). Disruption of this relay likely destabilizes bidirectional information flow between temporal and parietal systems, leading to impaired responsiveness. This interpretation is consistent with major theoretical models of consciousness, including the global neuronal workspace (GNW) theory, integrated information theory (IIT), and the network inhibition hypothesis (3, 8, 10–14). Collectively, these frameworks suggest that the posterior lateral temporal cortex is not an isolated center but a vulnerable relay node within distributed consciousness networks.

Although both APR − and APR + groups exhibited comparable interictal and ictal EEG profiles consistent with right MTLE, only the APR − group demonstrated significant hypometabolism in the posterior lateral temporal region. This dissociation highlights potential limitations of surface EEG in detecting subtle but clinically meaningful disruptions. FDG-PET, by revealing posterior temporal metabolic alterations associated with impaired responsiveness, provides complementary insights into consciousness-related circuitry not readily visible in electrophysiological recordings. Consistent with recent findings, impaired responsiveness may relate to reductions in EEG signal complexity rather than overt topographic changes (15), suggesting that posterior temporal hypometabolism could represent a metabolic correlate of hidden network disintegration.

While the posterior lateral temporal region was the only region to survive multiple-comparison correction, hypometabolic trends in posterior mesial and basal temporal regions suggest a broader temporal network vulnerability. This pattern aligns with evidence that mesial, basal, and lateral temporal structures jointly contribute to variability in semiology and responsiveness in TLE (16). These discrete metabolic profiles likely reflect a spectrum of network integrity, where preserved metabolism supports maintained responsiveness during seizures.

### Clinical implications

The distinctive pattern of posterior lateral temporal hypometabolism in APR − patients represents a potential neuroimaging biomarker of impaired responsiveness in right MTLE. Although our study lacks long-term cognitive and behavioral follow-up data, these metabolic signatures may serve as predictive markers for post-operative outcomes. Notably, our previous ictal SPECT study demonstrated parietal hyperperfusion in patients without preserved responsiveness (4), whereas the present FDG-PET analysis revealed posterior temporal hypometabolism. These complementary findings underscore the value of multimodal imaging in elucidating seizure-related network dysfunction (17).

### Limitations and future directions

Several limitations must be acknowledged. First, the retrospective design and modest cohort size (n = 49) may limit generalizability. Second, although our visual analysis reflects clinical practice, it may not detect subtle metabolic alterations that advanced quantitative methods could reveal. While ROI-based quantification is sometimes advocated, we did not adopt this approach as our main analytic strategy because results may vary substantially depending on subjective ROI placement. Instead, we attempted supplementary semi-automated analyses using BTX software (Seoul National Univ.) for SUVR quantification and Z-score maps generated by MIMneuro (MIM Software Inc., OH, USA) (18). However, these did not provide significant additional insights, largely because the available anatomical templates lacked sufficient resolution to reliably distinguish anterior and posterior temporal subregions. This limitation underscores the need for future quantitative methods with higher anatomical resolution, such as voxel-wise or surface-based analytic frameworks. Third, although our four-domain protocol parallels validated instruments, formal application of the Responsiveness in Epilepsy Scales (RES-II) or culturally adapted versions would improve cross-center comparability. Finally, multimodal studies integrating FDG-PET with connectivity-based methods (DTI, rs-fMRI) and prospective longitudinal outcome assessments will be crucial to validate posterior temporal hypometabolism as a biomarker of ictal responsiveness (19).

## Conclusion

This investigation indicates that impaired responsiveness in right MTLE is fundamentally linked to posterior lateral temporal dysfunction, with broader mesial and basal temporal vulnerability reflecting disruption of consciousness-supporting circuits. These metabolic signatures bridge theoretical models of consciousness with clinical epileptology and may serve as preliminary biomarkers for predicting responsiveness in right MTLE (2, 8, 10, 12–14, 16, 20).

## Data Availability

The raw data supporting the conclusions of this article will be made available by the authors, without undue reservation.
