# Supplementary material for: Preserved ictal responsiveness in right mesial temporal lobe epilepsy: metabolic correlates in posterior temporal networks with FDG-PET
Source: Front Neurol. 2025 Oct 23;16:1690510. doi: 10.3389/fneur.2025.1690510 (PMC12588851; doi:10.3389/fneur.2025.1690510)
Supplement: Supplementary file 1 [file Data_Sheet_1.docx]

**Supplementary Table 1. Comparison between APR classification in this study and domains assessed by the Responsiveness in Epilepsy Scales (RES-I and RES-II)**

| **APR classification (this study)** | **RES-I / RES-II domains** | **Correspondence and differences** |
| --- | --- | --- |
| **Verbal memory retention**  (word recall, immediate memory; e.g., recall of patient’s own name, remembering a presented color or number sequence for later retrieval) | RES-I: repetition and recall of words or numbers  RES-II: simplified memory item (e.g., recall of a presented word) | This domain directly corresponds to the memory domain in RES-I/II. |
| **Temporal-spatial orientation** (orientation to time and place; e.g., questions about current time, place, or setting) | RES-I: orientation items such as “Where are you now?” or “What is the year/month?”  RES-II: orientation questions simplified to reduce administration errors | Equivalent orientation domain. RES-II applies fewer items, improving feasibility and reducing error rates. |
| **Comprehension of verbal commands** (understanding spoken instructions; e.g., “Clap three times,” “Raise your right arm”) | RES-I: simple verbal commands (e.g., “Wave your hand”)  RES-II: standardized motor command comprehension | Closely corresponds. RES-II employs standardized verbal and motor commands, whereas our protocol used clinically standardized items. |
| **Execution of motor tasks** (performing motor commands; e.g., clapping the instructed number of times, raising the left or right arm as requested) | RES-I: motor responses (e.g., hand waving, tongue protrusion)  RES-II: simplified set of motor responses | Direct correspondence. RES protocols emphasize standardized motor responses, while our study relied on established EMU clinical practice. |

**Supplementary Table 2. Categorical distribution of FDG-PET hypometabolism severity (minimal, moderate, severe) between RMTLE patients with and without APR**

| **Regional hypometabolism** | **APR (-) Group (n=33)** | | | **APR (+) Group (n=16)** | | | | **p-value** |
| --- | --- | --- | --- | --- | --- | --- | --- | --- |
| **Degree of hypometabolism** | **Minimal** | **Moderate** | **Severe** | **Minimal** | **Moderate** | | **Severe** |  |
| **Anterior temporal regions** | | | | | | | | |
| **Temporal pole** |  |  |  |  | |  |  |  |
| Ipsilateral | 1(3.0%) | 18(54.5%) | 14(42.4%) | 2(12.5%) | | 9(56.3%) | 5(31.3%) | 0.25168 |
| Contralateral | 21(63.6%) | 12(36.4%) | -* | 11(68.8%) | | 5(31.3%) | -* | 0.72706 |
| **Basal temporal** |  |  |  |  | |  |  |  |
| Ipsilateral | -* | 16(48.5%) | 17(51.5%) | -* | | 11(68.8%) | 5(31.3%) | 0.18560 |
| Contralateral | 11(33.3%) | 20(60.6%) | 2(6.1%) | 7(43.8%) | | 9(56.3%) | -* | 0.32962 |
| **Mesial temporal** |  |  |  |  | |  |  |  |
| Ipsilateral | -* | 7(21.2%) | 26(78.8%) | -* | | 3(18.8%) | 13(81.3%) | 0.84267 |
| Contralateral | 2(6.1%) | 29(87.9%) | 2(6.1%) | 3(18.8%) | | 12(75.0%) | 1(6.3%) | 0.31237 |
| **Lateral temporal** |  |  |  |  | |  |  |  |
| Ipsilateral | -* | 20(60.6%) | 13(39.4%) | 2(12.5%) | | 12(75.0%) | 2(12.5%) | 0.01494 |
| Contralateral | 23(69.7%) | 10(30.3%) | -* | 13(81.3%) | | 3(18.8%) | -* | 0.39523 |
| **Posterior temporal regions** | | | | | | | | |
| **Basal temporal** |  |  |  |  | |  |  |  |
| Ipsilateral | -* | 18(54.5%) | 15(45.5%) | 3(18.8%) | | 11(68.8%) | 2(12.5%) | 0.00328 |
| Contralateral | 13(39.4%) | 18(54.5%) | 2(6.1%) | 7(43.8%) | | 9(56.3%) | -* | 0.54590 |
| **Mesial temporal** | | | | | | | | |
| Ipsilateral | -* | 12(36.4%) | 21(63.6%) | 1(6.3%) | | 12(75.0%) | 3(18.8%) | 0.00203 |
| Contralateral | 7(21.2%) | 24(72.7%) | 2(6.1%) | 9(56.3%) | | 7(43.8%) | -* | 0.03242 |
| **Lateral temporal** | | | | | | | | |
| Ipsilateral | 1(3.0%) | 24(72.7%) | 8(24.2%) | 6(37.5%) | | 10(62.5%) | -* | **0.00056** |
| Contralateral | 28(84.8%) | 4(12.1%) | 1(3.0%) | 16(100%) | | -* | -* | 0.12501 |
| **Extratemporal regions** | | | | | | | | |
| **Medial frontal** | | | | | | | | |
| Ipsilateral | 9(27.3%) | 19(57.6%) | 5(15.2%) | 4(25.0%) | | 12(75.0%) | -* | 0.47346 |
| Contralateral | 12(36.4%) | 20(60.6%) | 1(3.0%) | 6(37.5%) | | 10(62.5%) | -* | 0.79348 |
| **Dorsolateral frontal** | | | | | | | | |
| Ipsilateral | 6(18.2%) | 26(78.8%) | 1(3.0%) | 6(37.5%) | | 10(62.5%) | -* | 0.11729 |
| Contralateral | 30(90.9%) | 3(9.1%) | -* | 10(62.5%) | | 6(37.5%) | -* | 0.01714 |
| **Orbitofrontal** | | | | | | | | |
| Ipsilateral | 4(12.1%) | 24(72.7%) | 5(15.2%) | 2(12.5%) | | 12(75.0%) | 2(12.5%) | 0.84830 |
| Contralateral | 16(48.5%) | 17(51.5%) | -* | 9(56.3%) | | 7(43.8%) | -* | 0.61379 |
| **Inferior frontal** | | | | | | | | |
| Ipsilateral | 1(3.0%) | 27(81.8%) | 5(15.2%) | -* | | 14(87.5%) | 2(12.5%) | 0.97450 |
| Contralateral | 21(63.6%) | 11(33.3%) | 1(3.0%) | 9(56.3%) | | 7(43.8%) | -* | 0.78999 |
| **Insula** | | | | | | | | |
| Ipsilateral | 6(18.2%) | 19(57.6%) | 8(24.2%) | -* | | 14(87.5%) | 2(12.5%) | 0.71145 |
| Contralateral | 18(54.5%) | 15(45.5%) | -* | 8(50.0%) | | 8(50.0%) | -* | 0.76730 |
| **Thalamus** | | | | | | | | |
| Ipsilateral | 27(81.8%) | 6(18.2%) | -* | 12(75.0%) | | 3(18.8%) | 1(6.3%) | 0.35974 |
| Contralateral | 33(100%) | -* | -* | 33(100%) | | -* | -* | -* |
| **Basal ganglia** | | | | | | | | |
| Ipsilateral | 29(87.9%) | 4(12.1%) | -* | 12(75.0%) | | 4(25.0%) | -* | 0.25760 |
| Contralateral | 33(100%) | -* | -* | 15(93.8%) | | 1(6.3%) | -* | 0.15096 |
| **Parietal** | | | | | | | | |
| Ipsilateral | 2(6.1%) | 23(69.7%) | 8(24.2%) | 2(12.5%) | | 12(75.0%) | 2(12.5%) | 0.25624 |
| Contralateral | 14(42.4%) | 18(54.5%) | 1(3.0%) | 8(50.0%) | | 8(50.0%) | - | 0.51915 |
| **Occipital** | | | | | | | | |
| Ipsilateral | 30(90.0%) | 3(9.1%) | -* | 16(100%) | | -* | -* | 0.2195 |
| Contralateral | 32(97.0%) | 1(3.0%) | -* | 16(100%) | | -* | -* | 0.48623 |
